# Effect of alemtuzumab over sNfL and sGFAP levels in multiple sclerosis

**DOI:** 10.3389/fimmu.2024.1454474

**Published:** 2024-08-19

**Authors:** Raquel Sainz-Amo, Alexander Rodero Romero, Enric Monreal, Juan Luis Chico García, José Ignacio Fernández Velasco, Noelia Villarrubia, Jose Luis Veiga González, Susana Sainz de la Maza, Fernando Rodríguez Jorge, Jaime Masjuan, Lucienne Costa-Frossard, Luisa María Villar

**Affiliations:** ^1^ Neurology Department, Hospital Universitario Ramón y Cajal, La Red Española de Esclerosis Multiple, Red de Enfermedades Inflamatorias, Instituto de Salud Carlos III (ISCIII), Instituto Ramón y Cajal de Investigación Sanitaria, Madrid, Spain; ^2^ Immunology Department, Hospital Universitario Ramón y Cajal, La Red Española de Esclerosis Multiple, Red de Enfermedades Inflamatorias, Instituto de Salud Carlos III, Instituto Ramón y Cajal de Investigación Sanitaria, Madrid, Spain

**Keywords:** alemtuzumab, sNfL, sGFAP, multiple sclerosis, SiMoA

## Abstract

**Introduction:**

Alemtuzumab is a highly effective pulsed immune reconstitution therapy for multiple sclerosis (MS).

**Aim:**

To evaluate serum neurofilament light chain (sNfL) and serum glial fibrillary acidic protein (sGFAP) in patients with relapsing-remitting MS who have been treated with Alemtuzumab over the course of 2 years.

**Methods:**

This prospective study involved MS patients treated with Alemtuzumab at a referral MS center. Both sNfL and sGFAP were analyzed at baseline and then again at 6, 12, and 24 months post-treatment using the single molecule array (SiMoA) technique. We also recruited matched healthy controls (HCs) for comparison.

**Results:**

The study included 46 patients (with a median age of 34.2 [Interquartile range (IQR), 28.7–42.3] years, 27 of which were women [58%]) and 76 HCs. No differences in demographic characteristics were observed between patients and HC. The median disease duration was 6.22 (IQR, 1.56–10.13) years. The median annualized relapse rate before treatment was 2 (IQR, 1–3). At baseline, sNfL and sGFAP levels were higher in MS patients (median of 18.8 [IQR, 10.7–52.7] pg/ml and 158.9 [IQR, 126.9–255.5] pg/ml, respectively) when compared to HC (6.11 [IQR, 2.03–8.54] pg/ml and 91.0 [72.6–109] pg/ml, respectively) (p<0.001 for both comparisons). The data indicates that 80% of patients had high (≥10 pg/ml) sNfL values at baseline. We observed a significant decrease in sNfL levels at 6 (65%, p = 0.02), 12 (70.8%, p<0.001), and 24 (78.1%, p<0.001) months. sNfL reached similar levels to HC only after 24 months of Alemtuzumab treatment. During the follow-up period, no changes were identified in the sGFAP values.

**Conclusion:**

Alemtuzumab leads to the normalization of sNfL values in MS patients after 2 years of treatment, with no apparent effect on sGFAP values.

## 1 Introduction

Serum biomarkers have emerged as a useful tool in multiple sclerosis (MS), especially since the establishment of fourth-generation immune assays over the past decade (1, 2). Neurofilaments are cytoskeletal proteins whose release into CSF and blood is a quantitative measure of neuronal injury (3). Serum neurofilament light chain (sNfL) in MS has been validated as a biomarker for clinical and radiological inflammation and as a predictor of disease worsening (4–7).

Serum glial fibrillary acidic protein (sGFAP) is the main intermediate filament of astrocytes (8). sGFAP has been suggested as a biomarker to detect progressive MS and disability deterioration separate from inflammation (9–12). The combination of both biomarkers seems to enhance predictive accuracy (9, 10).

Alemtuzumab, a humanized monoclonal antibody, is a significant figure among the current pulsed immune reconstitution therapies. It works by binding to CD52, leading to a substantial reduction in autoreactive T and B-lymphocytes (13). This action paves the way for a new immune cell population, which is less likely to launch an immunological attack on the central nervous system (CNS) (14–17). The effectiveness of Alemtuzumab has been proven through rigorous clinical investigations, including phase II (CAMMS223) and phase III (CARE-MS I & II) trials, as well as observational studies with patients suffering from highly active disease (18–21).

The impact of Alemtuzumab on both sNfL and sGFAP levels has begun to be studied (22), but the evidence is limited.

We aimed to evaluate sNfL and sGFAP in patients with relapsing-remitting MS (RRMS) treated with Alemtuzumab over 2 years and to compare their values with a cohort of matched healthy controls (HCs). In addition, we intended to analyze the relationship between sNfL and sGFAP values and changes in disease course in terms of no evidence of disease activity-3 (NEDA-3), risk of relapse-associated worsening (RAW), and progression independent of relapse activity (PIRA).

## 2 Methods

### 2.1 Study design

This was an observational study with prospective data collection, aligning with the Strengthening the Reporting of Observational Studies in Epidemiology (STROBE) statement. Patients were recruited for the study at the Hospital Universitario Ramón y Cajal in Madrid, Spain. We consecutively enrolled patients with RRMS who began Alemtuzumab treatment between July 2015 and January 2022. These patients were followed until January 31, 2024. Both treatment-naïve and previously treated patients with one or more disease-modifying therapies (DMTs) were included. A wash-out period of four weeks was established for patients previously treated with monoclonal antibodies or fingolimod. No wash-out period was used for other drugs. Age, sex, and body mass index matched HCs were recruited between August 2023 and February 2024.

### 2.2 Standard protocols and patient consents

The study received approval from the Hospital Universitario Ramón y Cajal ethics committee, and all patients, along with HC, signed an informed consent prior to participation. Anonymized data, which support the findings of this study, will be available to any qualified investigator upon reasonable request for 3 years following the publication of the study.

### 2.3 Treatment

Patients received 60 mg of Alemtuzumab intravenously for five consecutive days. After 12 months, they were given a repeated dose of 36 mg intravenously over three consecutive days. Additional doses of 36 mg of Alemtuzumab were utilized at the signs of new disease activity, such as a relapse or the detection of new, enlarging, or contrast-enhancing MRI lesions. Premedication with 1000 mg of intravenous methylprednisolone, an oral antihistamine, paracetamol, and omeprazole was given. Furthermore, patients received prophylactic treatments with acyclovir and trimethoprim/sulfamethoxazole.

### 2.4 Data collection

Demographic, clinical, and radiological variables were collected at baseline. Experienced neurologists conducted all EDSS evaluations at least every 3 months and additional examinations in the event of a relapse. A baseline MRI was taken within a month prior to the start of treatment in accordance with established clinical protocols. Subsequent control MRI studies were done annually.

### 2.5 Sample collection

Patient blood specimens were collected just before initiating Alemtuzumab treatment and again at 6, 12, and 24 months after that. Serum sample aliquots were procured and stored at -80° until they were processed.

### 2.6 Serum sNfL and sGFAP quantification

sNfL and sGFAP were quantified using an SR-X instrument (Quanterix, Lexington, MA) with the single molecule array (SIMOA) technique (Quanterix, Billerica, MA). We employed the NF-light Advantage Kit (Quanterix, Billerica, MA) and the Serum GFAP Discovery Kit (Quanterix, Billerica, MA), respectively, following the manufacturer’s instructions. The mean inter- and intra-assay coefficients equaled 6% and 7% for sNfL and 6% and 10% for sGFAP, respectively. The research team handling the evaluation of the serum samples remained unaware of the clinical data.

### 2.7 Definitions

We applied the revised 2017 McDonald criteria for patient diagnosis (23). Disability was assessed using the EDSS score (24). Confirmed disability worsening was defined as an increase of at least 1.5 points in the EDSS if the baseline score was 0, a rise of at least 1 point if the previous EDSS was between 1 and 5, and a minimum 0.5 point increase for patients with a baseline EDSS of 5.5 or higher (25). NEDA-3 was defined as the absence of relapses, disability worsening, and new and/or enlarged T2 lesions or gadolinium-enhancing lesions on MRI (26). Conversely, patients experiencing either a relapse, MRI activity or an exacerbation of neurological disability were classified as having evidence of disease activity-3 (EDA-3). RAW and PIRA were defined as previously described (27).

The cut-off applied for sNfL and sGFAP levels was established at the 90th percentile value of the corresponding HC, which was 10 pg/ml for sNfL and 140 pg/ml for sGFAP, in line with the benchmarks used in previous studies (5, 7, 28).

### 2.8 Statistical analyses

Descriptive analyses were summarized using absolute and relative proportions for categorical variables, and differences were examined using either a χ² or Fisher’s exact test. The median with an interquartile range (IQR) was employed to describe continuous variables, and associations between groups were evaluated using the Kruskal-Wallis and Mann Whitney-U tests. We performed statistical analyses using the GraphPad Prism 9.0 software (GraphPad Prism Inc., San Diego, CA). All tests were two-tailed, and a significance level of P < 0.05 was deemed significant.

## 3 Results

**Figure 1:**
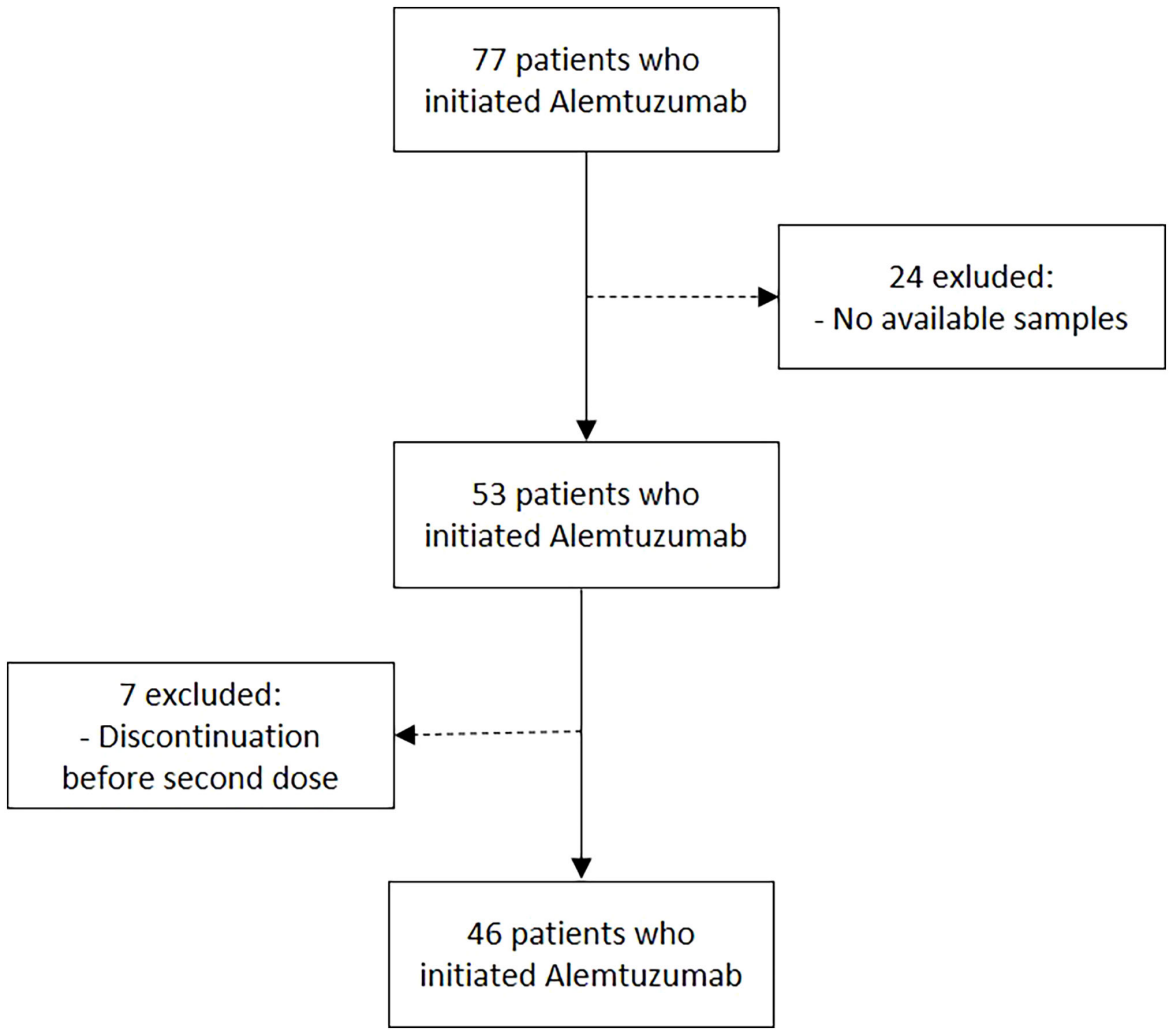
Flowchart of patients included.

We incorporated 46 patients (27 women (58%)) into the study, for a combined total of 77 (**Figure 1**), all of whom initiated Alemtuzumab in a single referral MS center. **Table 1** displays the clinical and demographic data of the cohort, as well as their matched HC.

**Table 1:** Baseline data.

	Patients (n=46)	Healthy Controls (n=76)	P value
Demographic characteristics			
Age (years)	34 [29– 42]	31 [26 – 46]	Ns
Sex (Female/Male)	27/19	47/29	Ns
BMI	24.2 [20.8 – 29.6]	22.5 [20.5 – 24.7]	Ns
Baseline Clinical, radiological, and laboratory data			
Disease duration (years)	6.2 [1.6 – 10.1]		
EDSS score	2.5 [1.5 – 4]		
ARR 1 year before treatment	2 [1 – 3]		
Previous treatment			
Naïve	12 (26.1%)		
Platform	1 (2.2%)		
Orals	24 (52.2%)		
Monoclonal Ab	9 (19.6%)		
T2 lesions (<10, 10-50, >50)	1 (2.2%), 27 (58,7%), 18 (39,1%)		
N. of Gd-enhancing lesions	1[0-4]		
N. of patients with Gd-enhancing lesions	24 (52.2%)		
Oligoclonal IgG bands	38/41 (92.7%)		
Oligoclonal IgM bands against lipids	24/38 (63.1%)		
sNfL [pg/mL]	18.8 [10.7 – 52.7]	6.11 [2 – 8.5]	p<0.0001
sGFAP [pg/mL]	158.9 [126.9 – 255.5]	91 [72.6 - 109]	p<0.0001

N, number of patients/controls; BMI, body mass index; EDSS, Expanded Disability Status Scale; ARR, annualized relapse rate; Ab, antibody; MRI, magnetic resonance imaging; sNfL, serum neurofilament light chains; sGFAP, serum glial fibrillary acidic protein.

Naïve: no previous treatment; Platform treatments included: interferon beta and glatiramer acetate; Oral drugs included: dimethylfumarate, fingolimod, and teriflunomide; Monoclonal Ab. included: natalizumab.

Categorical variables are shown as numbers (%). Continuous variables are described as median [IQR].

NS, non-significant.

The median age (IQR) of Alemtuzumab-treated patients was 34.2 (28.7–42.3) years. The disease duration at the onset of Alemtuzumab was 6.2 (1.6–10.1) years. Twelve patients (26.1%) were naïve, and 34 (73.9%) switched from other DMTs due to lack of efficacy (24, 52.2%) or safety concerns (10, 21.7%). The annualized relapse rate (ARR) the year prior to treatment initiation was 2 (1–3). Eighteen patients (39.1%) had more than 50 lesions in their baseline MRI, while 24 (52.2%) had gadolinium-enhancing lesions.

The values of sNfL and sGFAP were higher at baseline in MS patients compared to HC (p<0.001 for both comparisons).

**Figure 2:**
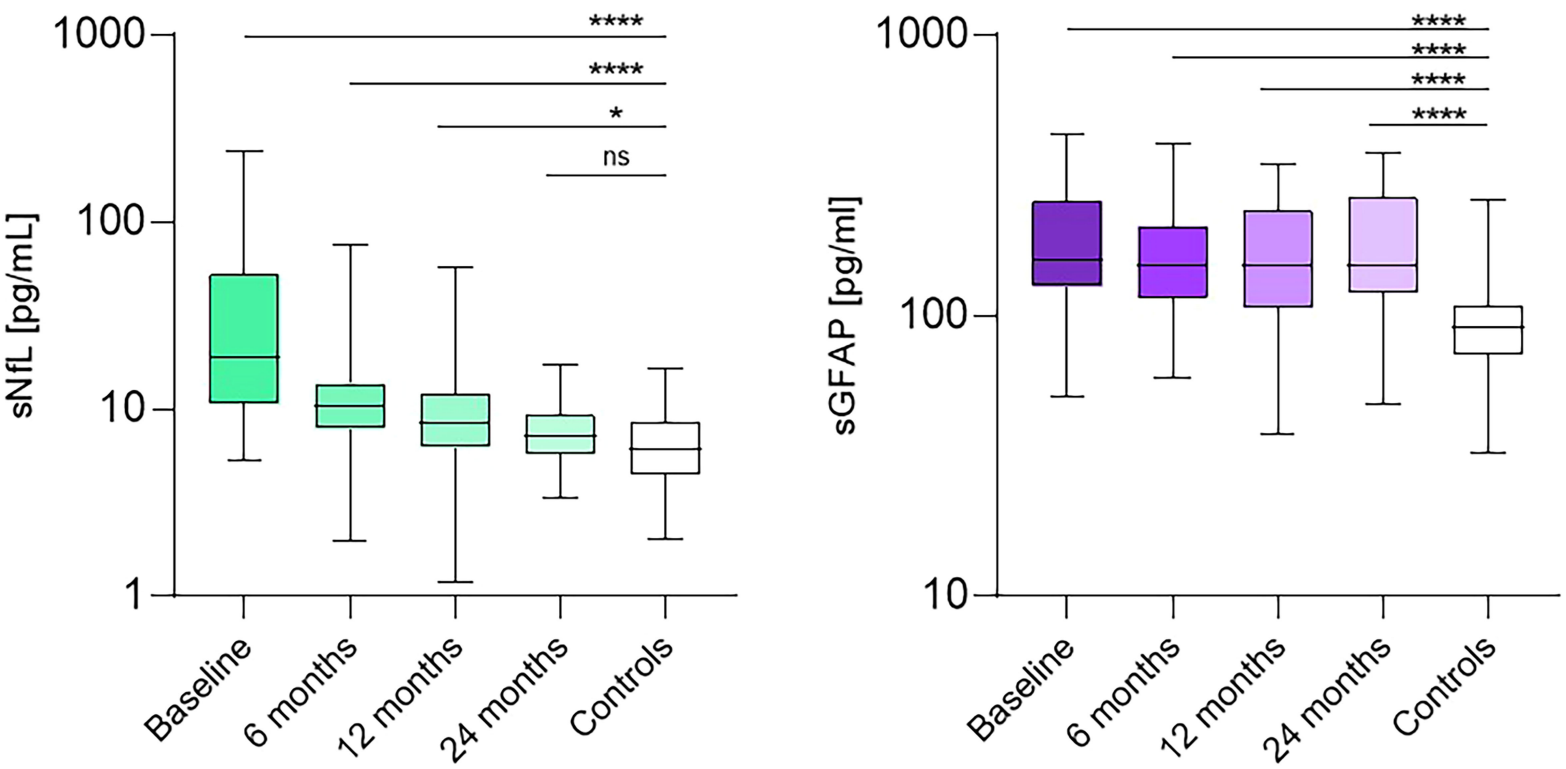
sNfL (pg/ml) values of controls and patients at baseline and after 6, 12, 24 months of treatment. sGFAP (pg/ml) values of controls and patients at baseline and after 6, 12, and 24 months of treatment. NS, non-significant, ****: p<0.0001, *: p<0.01.

A substantial decrease in sNfL levels was observed at 6 (65%, p = 0.02), 12 (70.8%, p<0.001), and 24 months (78.1%, p<0.001). sNfL levels were akin to those of the HC only after 24 months of Alemtuzumab treatment (**Figure 2**). No changes in sGFAP values were identified during the follow-up period (**Figure 2**).

We did not find differences in sNfL and SGFAP values between naïve and previously treated patients neither at baseline nor at 6, 12, 24 months. Likewise, we did not find differences between sexes (**Supplementary Table 1**).

Thirty-seven patients (80%) exhibited high (≥10 pg/ml) sNfL values at baseline. Patients presenting these high sNfL values had a higher number of gadolinium-enhancing lesions at baseline (median 1 [IQR 0 – 5] vs. 0 [0 – 0.75] p = 0.02).

Patients with high sNfL at the baseline had a greater number of new T2 lesions in the MRI performed after the first year of treatment (1 [0–2] vs. 0 [0-0], p=0.006). Similarly, NEDA-3 (44.2% vs. 88.9%, p=0.02) was achieved by a smaller proportion of these patients during the first year. However, these differences dissipated in the following years of follow-up (39.4% vs 78%, p=0.06 after second year).

**Figure 3:**
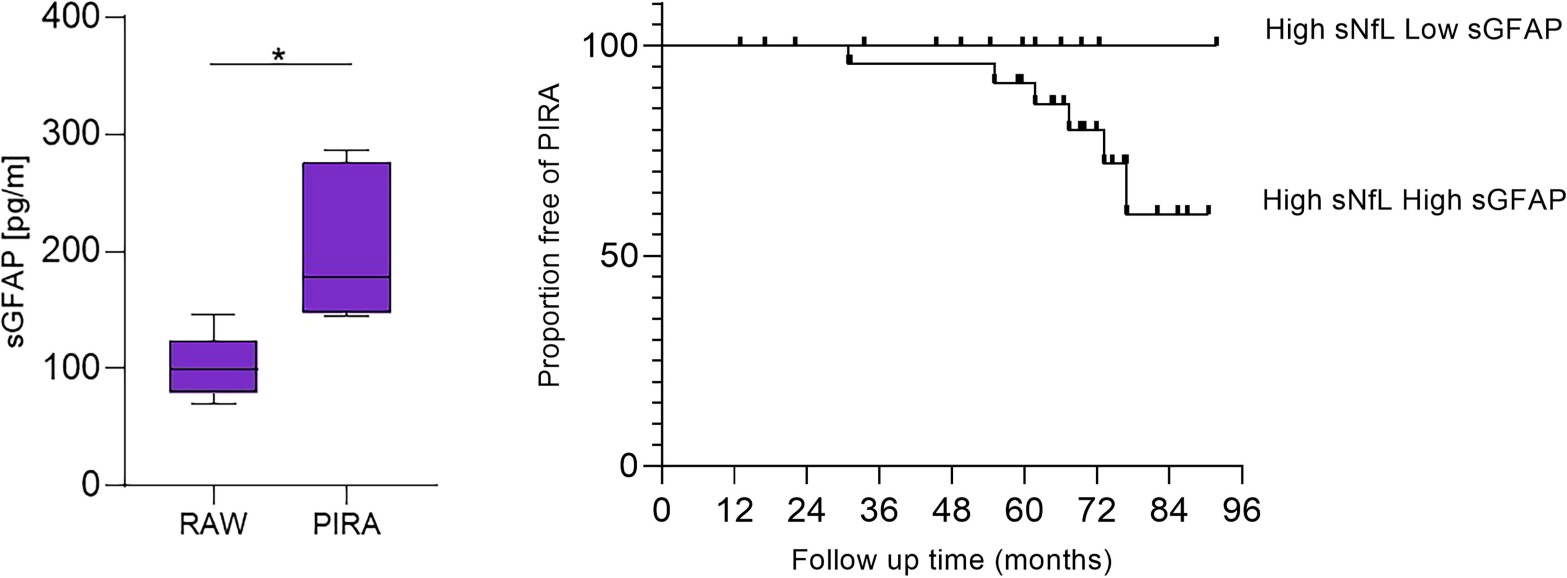
Comparison of baseline sGFAP (pg/ml) values in patients with high sNfL (pg/ml). Kaplan-Meier comparing time to PIRA in patients with high sNfL (pg/ml) who had high versus low sGFAP (pg/ml). *: p=0.02.

We next analyzed the risks associated with RAW and PIRA. Median [IQR] follow-up time was 5.8 years [4.8-7.7], 76% of patients were followed up for at least five years. No significant differences were noted between patients with high sNfL at baseline and those with lower values concerning the risk of RAW (HR 0.3 [0.04 – 2.1], p=0.2). However, throughout the follow-up, no patients with low sNfL experienced either RAW or PIRA. In contrast, for the 27 patients with high sNfL who were followed 5 years post-Alemtuzumab administration, the cumulative incidences of RAW and PIRA were 15% and 14.4%, respectively. Baseline sGFAP levels served as a differentiating factor for patients who encountered RAW during follow-up, as they exhibited lower values compared to those suffering from PIRA (99 [79–123] vs. 178 [147–276], p = 0.02). Furthermore, patients with higher sGFAP values demonstrated a tendency for an increased risk of PIRA over time (HR 3.7 [0.5–27.5], p = 0.2) (**Figure 3**).

## 4 Discussion

sNfL levels indicate acute axonal damage and are associated with acute inflammatory activity seen as clinical relapses or T2-weighted or contrast-enhancing lesions (29). These levels can be used as a biomarker for monitoring inflammation (5) and disease progression (7). Combining sNfL levels with sGFAP might enhance the ability to identify patients at risk of disease progression (10).

The early administration of high-efficacy DMTs (HE-DMTs) in patients with high sNfL levels has been associated with no disability worsening (7). However, there are only a few studies focused on the analysis of sNfL values over time in patients who have started on a specific HE-DMT. Additionally, there are even fewer studies that analyze both sNfL and sGFAP.

We aimed to explore the role of both biomarkers in a cohort of highly active patients treated with Alemtuzumab. First, we analyzed sNFL and sGFAP over 2 years and compared the values to a cohort of matched HCs. We observed that sNfL decreased progressively, as described in other cohorts (22, 30–33), but did not reach similar values to those of the HC until a follow-up of two years. A reduction in sNfL was also described two years after alemtuzumab initiation in other cohort of patients treated with alemtuzumab who showed moderate levels at treatment onset (22). We could show that results are similar in a more active cohort with clearly higher baseline sNfL values. Likewise, a clear decrease of sNfL was observed in the Alemtuzumab arm of the CARE-MS I study (27), this decrease was higher compared with patients treated with Interferon-beta-1a. In our cohort we also see a decrease in sNfL values until the normalization is reached and there is no difference with HCs. Higher baseline sNfL values were associated with higher T2 lesion load, as we describe in our cohort.

By contrast, in patients with high sGFAP levels at treatment initiation these levels remained stable over time and were higher than those of HC, which confirms previous findings in which symptomatic controls were used as control group (22).

Alemtuzumab eliminates the abnormal B and T cells (13) and let the immune system to reconstitute a normal lymphocyte response. This seems to work very efficiently in patients who have not an intrathecal activation of the innate immune response, reflected by the high sGFAP values. By contrast, in patients with increased sGFAP values a trend to a higher risk of PIRA was observed, despite the normalization of sNfL. The association of sGFAP with high risk of disability progression was already described (10). Our data strongly suggest that normalization of sGFAP values it crucial for avoiding disease progression in MS.

We examined whether baseline sNfL was associated with an increase in disability over time. We set a cut-off value of 10 pg/mL for sNfL and 140 pg/mL for sGFAP based on the 90th percentile of our population of matched HCs. Patients with elevated sNfL displayed more clinical and radiological activity during the initial year. However, these differences were not apparent in the following years. Along with the normalization of sNfL, this information emphasizes the effectiveness of Alemtuzumab in managing axonal damage and inflammation.

No patient with low sNfL values demonstrated RAW or PIRA during follow-up. Additionally, high levels of sGFAP in this patient group were associated with a trend toward a higher risk of PIRA. These data suggest that astrogliosis (34) may remain active despite Alemtuzumab treatment and contribute to patient disability worsening independent of relapses.

The primary limitation of our study was the reduced sample size. These findings should be validated in larger, multicenter cohorts.

In conclusion, we observed a normalization of sNfL levels at follow-up, while sGFAP concentrations remained unchanged. This suggests that Alemtuzumab reduces acute axonal damage but has little or no effect on astrogliosis.

## Data Availability

The raw data supporting the conclusions of this article will be made available by the authors, without undue reservation.
