# Supplementary material for: Effect of alemtuzumab over sNfL and sGFAP levels in multiple sclerosis
Source: Front Immunol. 2024 Aug 19;15:1454474. doi: 10.3389/fimmu.2024.1454474 (PMC11366608; doi:10.3389/fimmu.2024.1454474)
Supplement: Supplementary file 1 [file Table1.docx]

**Supplementary table 1.** **Comparison of sNfL and sGFAP at baseline, 6, 12, 24, months between Naïve vs previously treated patients, and female vs male patients.**

| **Naïve vs previously treated patients** | | | | | | |
| --- | --- | --- | --- | --- | --- | --- |
| sNfL (pg/ml) | | | | sGFAP (pg/ml) | | |
|  | Naive | Previously treated | P value | Naive | Previously treated | P value |
| Baseline | 16.5 [10.1 – 40.5] | 29.1 [16.4 – 53.5] | 0.2 | 173.7 [132.4 – 241.6] | 151.6 [121.4 – 291] | 0.8 |
| 6 months | 10.1 [5.1 – 11.7] | 10.4 [7.8 – 13.8] | 0.4 | 164 [118.1 – 190] | 150 [114 – 220] | 0.9 |
| 12 months | 6.3 [4.8 – 8.9] | 9.6 [6.5 – 12.3] | 0.6 | 137.8 [113 – 199.4] | 165.6 [100.4 - 240.9] | 0.7 |
| 24 months | 6.6 [5.2 – 7.2] | 7.6 [5.6 – 11.1] | 0.2 | 149 [137 – 200] | 150.7 [111.4 – 281.2] | 0.8 |
| **Female vs male patients** | | | | | | |
| sNfL (pg/ml) | | | sGFAP (pg/ml) | | |  |
|  | Female patients | Male patients | P value | Female patients | Male patients | P value |
| Baseline | 18.5 [10.6 – 53.2] | 24.4 [11 – 52.4] | >0.9 | 169.6 [141 – 313] | 144 [144 – 182] | >0.9 |
| 6 months | 9.9 [7.9 – 12.8] | 10.7 [7.2 – 13.8] | >0.9 | 164 [128 vs 254.8] | 124 [105.8 – 202.8] | >0.9 |
| 12 months | 8.2 [5.1 – 12.3] | 9.4 [6.1 – 12.1] | >0.9 | 174.7 [122.7 – 236] | 127.6 [89.7 – 245.1] | >0.9 |
| 24 months | 6.9 [5.6 – 9.1] | 7 [5.5 – 10.1 | >0.9 | 151.4 [122.7 – 263.4] | 147.5 [ 111.4 – 303.6] | >0.9 |

Footnote: Abbreviations: M, months; sNfL, serum neurofilament light chains; sGFAP, serum glial fibrillary acidic protein.

Continuous variables are described as median [IQR].
